# Mitochondrial iron-sulfur cluster biogenesis from molecular understanding to clinical disease

**DOI:** 10.17712/nsj.2017.1.20160542

**Published:** 2017-01

**Authors:** Majid Alfadhel, Marwan Nashabat, Qais Abu Ali, Khalid Hundallah

**Affiliations:** *From the Department of Pediatrics, King Abdulaziz Medical City, Ministry of National Guard-Health Affairs, Riyadh, Kingdom of Saudi Arabia*

## Abstract

Iron–sulfur clusters (ISCs) are known to play a major role in various protein functions. Located in the mitochondria, cytosol, endoplasmic reticulum and nucleus, they contribute to various core cellular functions. Until recently, only a few human diseases related to mitochondrial ISC biogenesis defects have been described. Such diseases include Friedreich ataxia, combined oxidative phosphorylation deficiency 19, infantile complex II/III deficiency defect, hereditary myopathy with lactic acidosis and mitochondrial muscle myopathy, lipoic acid biosynthesis defects, multiple mitochondrial dysfunctions syndromes and non ketotic hyperglycinemia due to glutaredoxin 5 gene defect. Disorders of mitochondrial import, export and translation, including sideroblastic anemia with ataxia, EVEN-PLUS syndrome and mitochondrial complex I deficiency due to nucleotide-binding protein-like protein gene defect, have also been implicated in ISC biogenesis defects. With advances in next generation sequencing technologies, more disorders related to ISC biogenesis defects are expected to be elucidated. In this article, we aim to shed the light on mitochondrial ISC biogenesis, related proteins and their function, pathophysiology, clinical phenotypes of related disorders, diagnostic approach, and future implications.

Iron sulfur clusters (ISCs) were first described in the early 1960s by Helmut Beinert.^1^-^3^ Subsequent research highlighted the crucial role of these proteins in different biological cellular processes in plants, prokaryotes and eukaryotes and their link to human diseases.^4^ The first human disease related to ISCs biogenesis pathway was Friedreich ataxia (FRDA) which was described in the 1860s. Since the elucidation of the molecular basis causing FRDA in 1996,^5^ several studies have been conducted to explore the types of mutations and the exact role of the mitochondrial membrane protein frataxin and its involvement in ISC pathway.^6^-^8^ More recently, with the application of advanced molecular diagnostic technologies along with whole exome sequencing, additional human disorders have been unveiled beyond FRDA. In this article, we aim to shed the light on mitochondrial ISC biogenesis, related proteins and their function, pathophysiology clinical phenotypes of related disorders, diagnostic approach, and future implications.

## Iron-sulfur proteins: biological function and relevance

Iron sulfur clusters are known to play a major role in various protein functions. Located in the mitochondria, cytosol, endoplasmic reticulum and nucleus, they contribute to respiration, iron homeostasis, heme biosynthesis, oxidative phosphorylation, citric acid cycle, and DNA replication and repair, among regulation of other pathways.^5^-^7^

Assembly of ISCs usually begins in mitochondria, where several other proteins essential for iron-sulfur (Fe-S) components maturation are found. There, ISCs are involved in enzymatic reactions of aconitase 1 and 2 of citric acid cycle, electron transfer of complexes I, II and III, fatty acid oxidation specifically electron-transfer-flavoprotein-ubiquinone oxidoreductase, biotin and lipoic acid,^9^ which is a fundamental cofactor involved in many cellular pathways. Iron-sulfar (Fe-S) components continue their maturation in mitochondria till they reach their target apoproteins, a process that’s been well investigated in prokaryotes and human equivalents.^10^

There are approximately 20 different proteins involved in the mitochondrial ISC biogenesis. These include: The sulfur donor nitrogen fixation gene 1,^11^ scaffold proteins such as Fe-S cluster scaffold (ISCU),^12^ LYR motif-containing protein 4 (LYRM4)^13^ - a eukaryotic specific accessory factor- required for cysteine desulfurase activity, Frataxin (FXN)^8^ as cysteine desulfurase depressor, Ferredoxins (FDX1, FDX2)^14^ and Ferredoxin reductase (FDXR)^15^ which are necessary for electron transport. Moreover, Fe-S cluster assembly 1(ISCA1),^16^,^17^ iron-sulfur cluster assembly 2 (ISCA2), and iron-sulfur cluster assembly factor for biotin synthase- and aconitase-like mitochondrial proteins, with a mass of 57kDa (IBA57)^17^ are also implicated in [4Fe-4S] assembly. In addition, there are molecular chaperone such as Mortalin (heat shock protein family A (Hsp70) member 9 (HSPA9),^18^,^19^ HSC20 gene^20^ that binds target proteins containing the LYR motif, an intermediate carrier glutaredoxin 5 (GLRX5),^21^ iron-sulfur cluster scaffold (NFU1)^22^ and bolA family member 3 (BOLA3)^23^,^24^ which are dedicated targeting factors for lipoic acid synthase,^25^ nucleotide-binding protein-like protein (NUBPL) - essential for complex I assembly-,^26^ ATP-binding cassette subfamily b, member 7 (ABCB7) that exports a sulfur containing compound from mitochondria,^27^ and growth factor, ERV1-like.^28^

## Pathophysiology

Mitochondrial ISCs process is crucial for the biosynthesis of mitochondrial, cytosolic and nuclear Fe-S containing proteins.^29^-^31^ In eukaryotes, ISCs are present in 2 forms: [2Fe-2S] rhombic, and [4Fe-4S] cubane.^32^

**Figure 1:**
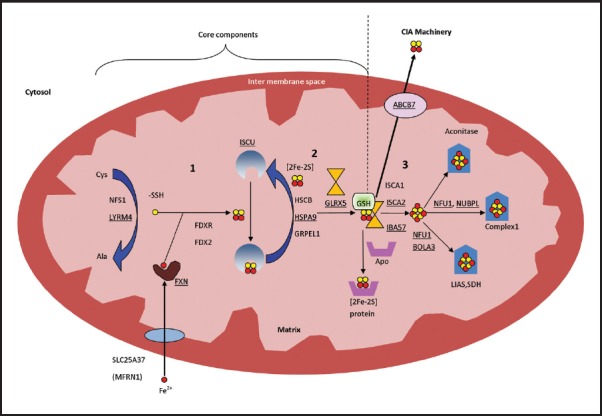
- Mitochondrial iron-sulfur cluster assembly: all involved proteins are written in their genetic names. Yellow circle presents sulfur and red circle presents iron. Numbers indicate the steps of the process; number 1 and 2 include the core components of the process. Proteins associated with known medical conditions are underlined. Ala - Alanine, BOLA3 - bolA family member 3, Cys - Cysteine, FDX2 - Ferredoxin 2, FDXR - Ferredoxin reductase, FXN - Frataxin, GLRX5 - glutaredoxin 5, GRPEL1 - GrpE like 1, GSH - tripeptide glutathione, HSCB - HscB mitochondrial iron-sulfur cluster cochaperone, HSPA9 - heat shock protein family A (Hsp70) member 9, IBA57 - iron-sulfur cluster assembly factor for biotin synthase- and aconitase-like mitochondrial proteins, with a mass of 57kDa, ISCA1 - iron-sulfur cluster assembly 1, ISCA2 - iron-sulfur cluster assembly 2, ISCU - Iron–sulfur cluster scaffold homolog, LIAS - lipoic acid synthetase, LYRM4 - LYR motif containing 4, NFS - Cysteine desulfurase, NFU1 - NFU1 iron-sulfur cluster scaffold, NUBPL - nucleotide binding protein like, SDH - succinate dehydrogenase.

The process is composed of three sequential steps (**Figure 1**); the first step starts by sulfur donation from cysteine desulfurase (NFS), which in turn forms a complex with the stabilizing protein LYRM4, to complete the reaction and release sulfane (-SSH).^33^,^34^ Simultaneously, iron is imported into the mitochondria through the inner membrane protein, SLC25A37 (Mitochondrial Iron Transporter 1).^35^ Meanwhile FXN probably (as there is no clear evidence) acts as iron chaperone to deliver the imported iron to scaffold protein ISCU and regulates the above mentioned cysteine desulfurase reaction.^36^,^37^ A reduction reaction then takes place by FDX2 and FDXR to reduce sulfane to sulfide, after which Fe-S assembly is accomplished on ISCU.^14^,^38^,^39^ In the second step, a series of proteins, HSC20, HSPA9 and GrpE like 1, react with the Fe-S loaded ISCU leading to detachment of [2Fe-2S] cluster.^20^,^40^ Monothiol GLRX5 then binds transiently to the released [2Fe-2S], in coordination with tripeptide glutathione to form a glutathione-containing complex^41^,^42^ and finally transfer the mature [2Fe-2S] cluster to apoproteins or export it outside the mitochondria, through the inner membrane protein ABCB7,^27^ to take part in the cytosol iron assembly (CIA) machinery. The aforementioned proteins are essential for the synthesis of all cellular ISC proteins, hence called the core components of ISC machinery.^9^ In the third and final step, A-type proteins ISCA1, ISCA2, and IBA57 carry out the maturation of all cellular [4Fe-4S] clusters and carry them to their apoproteins either directly or through other specific proteins, like NFU1, NUBPL, and BOLA3 (**Figure 1**), which contribute to the maturation of specific [4Fe-4S] containing proteins.^17^ Further details of the process can be reviewed in references.^9^,^43^

## Classification of mitochondrial ISC biogenesis defects

The ISCs are fundamental for many cellular pathways. Therefore, it is not unexpected that impairment of this pathway will cause a wide variety of diseases in humans (**Table 1**). Depending on the step of the pathway where there is a defect, disorders are classified into 3 categories:

1) Iron-sulfur assembly defects which include 5 disorders: Friedreich ataxia due to FXN gene defect, combined oxidative phosphorylation deficiency 19 due to LYRM4 gene defect, infantile complex II/III deficiency (IMC23D) due to NFS1 gene defect, hereditary myopathy with lactic acidosis due to ISCU gene defect and mitochondrial muscle myopathy due to Ferredoxin 1-Like protein (FDX1L) gene defect.
2) Lipoic acid biosynthesis defects which include 5 disorders: Multiple mitochondrial dysfunctions syndrome 1 due to NFU1 gene defect, multiple mitochondrial dysfunctions syndrome 2 due to BOLA3 gene defect, multiple mitochondrial dysfunctions syndrome 3 due to IBA57 gene defect, multiple mitochondrial dysfunctions syndrome 4 due to ISCA2 gene defect and non ketotic hyperglycinemia due to GLRX5 gene defect.
3) Disorders of mitochondrial import, export and translation: Sideroblastic anemia with ataxia due to ABCB7 gene defect, EVEN-PLUS syndrome due to HSPA9 gene defect and, mitochondrial complex I deficiency due to NUBPL gene defect.

**Table 1:** Diseases caused by mitochondrial iron-sulfur cluster biogenesis.

Variables	Frataxin (FXN)	ISD11 (LYRM4)	NFS1	ISCU	FDX1L	GLRX5	NFU1	BOLA3	IBA57	ISCA2	NUBPL	ABCB7	HSPA9
OMIM#	22930	615595	603485	255125	614585	616860 and 616859	605711	614299	615330	616370	613621	301310	616854
Disease	Friedreich ataxia (FRDA)	Combined oxidative phosphorylation deficiency 19	Infantile complex II/III deficiency (IMC23D)	Myopathy with lactic acidosis, hereditary	mitochondrial muscle myopathy	616860: Anemia, sideroblastic, 3, pyridoxine-616859: refractory Spasticity, childhood-	Multiple mitochondrial dysfunctions syndrome 1	Multiple mitochondrial dysfunctions syndrome 2	Multiple mitochondrial dysfunctions syndrome 3	Multiple mitochondrial dysfunctions syndrome 4	Mitochondrial complex I deficiency	Sidroblastic anemia with ataxia	Even-plus syndrome
Year	1996	2013	2014	2008	2014	2007 and 2011	2011	2011	2013	2015	2010	1999	2015
Pathway defect	Core [Fe-S] assembly	Core [Fe-S] assembly	Core [Fe-S] assembly	Core [Fe-S] assembly	Core [Fe-S] assembly	[Fe-S] transfer to specific recipients	[Fe-S] transfer to specific recipients	[Fe-S] transfer to specific recipients	[Fe4-S4] assembly	[Fe4-S4] assembly	Mitochondrial translation; complex I assembly	Mitochondrial export	Mitochondrial iron import
Number of patients/Prevalence	1:50000	2	3	25	1	5	20	3	2	6	7	22	3
Age of onset	Childhood-Adult (usually 2nd decade)	Neonatal	Infantile	Childhood	Childhood	Adult and childhood	neonatal and infantile	Infantile	Neonatal	Infantile	Infantile	Childhood	Prenatal
Origin	Panethnic	Lebanon and Syria	Canada	Sweden and Norway	Morocco	Italy and China	Mexico, Germany, Serbia, Romania, Pakistan	India, Australia, Africa	Morocco	Saudi	Argentina, Germany, Canada, Australia, Netherlands	USA	Chile and Korea
Clinical hints	Ataxia, dysarthria, muscle weakness, spasticity in the lower limbs, scoliosis, bladder dysfunction, absent lower limb reflexes, and loss of position and vibration sense, cardiomyopathy, DM	Hypotonia, respiratory distress	Hypotonia, respiratory distress, seizure, multisystem organ failure	Muscle weakness, exercise intolerance and cardiomyopathy	Severe proximal lower limb weakness and muscle cramps	616860: Sideroblastic anemia, hepatosplenomegaly and jaundice 616859: Spastic paraplegia, spinal lesion, and optic atrophy	Hypotonia, respiratory distress, seizure, Neurologic regression pulmonary hypertension, lethargy, poor feeding, White matter lesions seen on brain imaging	Hypotonia, respiratory distress, seizure, Neurologic regression, lethargy, poor feeding, optic atrophy, white matter lesions seen on brain imaging	Severe hypotonia, generalized muscle weakness, absent primitive reflexes, microcephaly and dysmorphic features (retrognathia, high palate, widely spaced nipples, arthrogryposis, cerebral atrophy and polymicrogyria on Brain MRI	Neuroregression, developmental delay, nystagmus with optic atrophy and diffuse white matter disease of the brain and spine	Hypotonia, muscle weakness, muscle atrophy exercise intolerance Muscle biopsy shows abnormal mitochondria, developmental delay, neuroregression, seizure, white matter lesions seen on brain imaging	Sidroblastic anemia and ataxia	EVEN-PLUS syndrome is characterized by short stature, vertebral and epiphyseal changes, microtia, midface hypoplasia with flat nose and triangular nares, cardiac malformations, and other findings including anal atresia, hypodontia, and aplasia cutis. The features overlap those reported in patients with CODAS syndrome
Biochemical hints	None	Lactic acidosis, metabolic acidosis, high liver enzymes low complexes I-IV in the muscle	Lactic acidosis, metabolic acidosis, high CK level and high liver enzymes, DIC picture, low complexes II and III in the muscle	Lactic acidosis, Myoglobinuria Histopathology showed succinate dehydrogenase and cytochrome c oxidase (COX) deficienc	Lactic acidosis, myoglobinuria and low complexes I, II and III in the muscle	616860: hypochromic microcytic anemia, increase ferritin level, ringed sideroblasts on bone marrow aspirate	Hyperglycinemia, metabolic acidosis, lactic acidosis, Increased urinary 2-hydroxybutyrate, Decreased activity of pyruvate dehydrogenase complex, low complexes I and II in the muscle	same as NFU1 gene defect	Hyperglycinemia, metabolic acidosis, lactic acidosis	Hyperglycinemia, metabolic acidosis, lactic acidosis	hypoglycemia, lactic acidosis, low complex I in the muscle	Increased free erythrocyte protoporphyrin, hypochromic microcytic anemia, ringed sideroblasts on bone marrow aspirate	Not specific
Mutation reported	90 % have expanded GAA repeat in intron 1 of FXN gene*	Missense mutation c.203G>T, p.Arg68Lys	Missense mutation c.251G>A, p.Arg72Gln	Splicing defect IVS5 + 382G>C, heterozygosity for the splicing defect and the missense mutation c.149G>A, p.Gly50Glu	homozygous mutation c.1A>T	616860: A> G homozygous transition 616859: Homozygous deletion c.151_153delAAG, p.K51del or compound heterozygosity for p.K51del and 8bp insertion	A homozygous missense mutation, c.545G>A(p.Arg182Gln), compound heterozygous for aforementioned mutation and a splice-site (c.545+5G>A) mutation, compound heterozygous mutation (g.69400462C>A, p.Gly208Cys); g.69592691_ 69648327del, [?]) compound heterozygous mutation (c.544C>T, [?], p.Arg182Trp);[?]), (c. 565G>A, p. Gly189Arg);[568G>A],;[Gly190 Arg]), (c.[544C>T];[?], p.[Arg182Trp];[?]), homozygous frameshift mutation c.302+3A>G (p.Val56Glyfs*9), compound heterozygous mutation (c.62G>C, p.Arg21Pro); (c.622G>T, p.Gly208Cys)	(c.136C4T, p.R46X)	(c.941A > C, p.Gln314Pro)	(c.229G>A, p.Gly77Ser)	Homozygous missense mutation (c.166G>A, p.Gly56Arg), intronic mutation: c.815-27T>C or compound heterozygous for (c.166G>A, p.Gly56Arg) and other mutation	Homozygous missense mutation(c.1200T>G(p.Ile400Met), Other several mutations near to or in transmembrane domains of the ABC transporter	Compound hgetrozygous mutation (c.383A > G (p.Y128C) and c.882_883delAG (p.V296*), homozygous missense mutation (c.376C > T;p.Arg126Trp).
Mortality	The average age of death was at 37.5 years (range, 5–71 years)	1/2	2/3	None	None	None	20/20	3/3	2/2	4/6	None	None	None

## Clinical phenotypes

### 1. Friedreich ataxia (OMIM#229300)

Inherited as an autosomal recessive disorder and considered as the most common spinocerebellar degenerative disease, with prevalence of 1:20,000-1:50,000. Friedreich ataxia is characterized by the triad: ataxia, areflexia and positive plantar response. Additional features include cardiomyopathy, diabetes mellitus, visual loss and deafness. Cognitive function and intelligence on the other hand are usually preserved.

Friedreich ataxia presents during childhood or adolescence. Clinically, ataxia is usually the first sign often followed by pyramidal signs, dysarthria and upper-limb ataxia. Areflexia and distal sensory loss are also present in most cases, and spasticity which occurs later can lead to discomfort, pain and contractures. Cardiac involvement is initially clinically asymptomatic. However, hypertrophic cardiomyopathy usually develops after the neurological symptoms. Approximately 30% of patients with FRDA develop diabetes mellitus.

Ophthalmological findings which present early on include fixation instability (square wave jerks), nystagmus, and blindness at a later age. Auditory neuropathy often leads to hearing abnormalities. Other features include mild dysphagia which progresses with disease advancement, skeletal abnormalities (scoliosis, and foot deformities such as pes cavus and talipes equinovarus).

Diagnosis of FRDA is made by combining clinical findings and molecular testing. Approximately 90% of cases of FRDA are homozygous for a disease causing GAA repeat expansion in intron 1 of FXN gene encoding frataxin, while 10% are compound heterozygotes for a disease causing GAA repeat expansion in one allele and another intragenic pathogenic variant in the other allele. The disease causing alleles exhibit expansion from 66 to 1700 GAA repeats, with most patients having between 600 and 1200 GAA repeats. Treatment is usually supportive but without a cure. Therefore prognosis has improved, however morbidity and quality of life remain a major concern. Cardiac complications including arrhythmias (especially atrial fibrillation) and congestive heart failure are the main causes of death. Early age of onset and other comorbidities, including diabetes mellitus, contribute to a survival age of around 40 years old.^44^-^49^

### 2. Combined oxidative phosphorylation deficiency type 19 due to LYRM4 gene defect (OMIM# 615595)

Combined oxidative phosphorylation deficiency is one of the most common enzymatic defects in patients with mitochondrial disorders, contributing up to 30% of all cases.^50^ To date, there are approximately 30 different types of defects, each classified according to the gene defect. Combined oxidative phosphorylation deficiency type 19 due to defect in LYRM4 gene is an autosomal recessive disease that was first described by Lim et al^51^ 2013 in 2 double first cousins with neonatal onset of hypotonia, respiratory distress, stridor, failure to thrive, poor feeding and hepatic steatosis. Biochemical workup showed lactic acidosis, metabolic acidosis, high liver enzymes, and low complexes I-IV in muscle and liver samples. Diagnosis was confirmed by identifying homozygous missense mutation in LYRM4 gene (c.203G>T, p.Arg68Leu).^51^ One of these 2 relatives died at 12 weeks of age while the other was reported healthy at the age of 20 years old.^51^

### 3. Infantile mitochondrial complex II/III deficiency due to NFS1 gene defect (OMIM#603485)

Farhan et al^52^ 2014 first described this autosomal recessive disease in 3 affected siblings to consanguineous parents from Old Order Mennonite community in Canada.^52^ These patients presented with hypotonia, respiratory distress, seizures, and multisystem organ failure. Biochemical workup revealed lactic acidosis, metabolic acidosis, high CK levels, high liver enzymes, disseminated intravascular coagulation features, and low complexes II and III in muscle sample. Diagnosis was confirmed through autozygosity mapping and whole exome sequencing, which identified homozygous missense mutation (c.215G>A, p.Arg72Gln) in NFS1 gene in all three affected siblings. Two of the 3 reported siblings died at 7 month of age due to cardiac failure.^52^

### 4. ISCU myopathy, also known as hereditary myopathy with lactic acidosis or as Swedish myopathy due to ISCU gene defect (OMIM#255125)

Inherited in an autosomal recessive pattern, these patients classically present with muscular manifestations including: myopathy, exercise intolerance, and premature exertional muscle weakness where muscles become hard, painful and tender during exercise. Laboratory investigations show mitochondrial respiratory chain complex I, II, and III defect, rhabdomyolysis, abnormal mitochondria in muscle biopsy, subsarcolemmal mitochondrial and lipid droplet accumulation, abnormal iron deposition, and decreased muscle succinate dehydrogenase and muscle mitochondrial aconitase.^53^-^56^

### 5. Mitochondrial muscle myopathy due to FDX1L gene defect (OMIM#614585)

It was reported only once, in a Jewish Moroccan female adolescent to consanguineous parents, who presented with severe proximal lower limb myopathy, muscle cramps and lactic acidosis. Biochemical workup showed lactic acidosis, myoglobinuria and low complexes I, II and III in the muscle. Notably, subsequent annual cardiac evaluation remained normal.^57^

### 6. Mitochondrial complex I deficiency due to NUBPL gene defect (OMIM# 613621)

Respiratory chain deficiency of complex I is the most common cause for mitochondrial disorders, accounting for approximately one third of all reported patients.^58^ As was recently demonstrated, NUBPL plays an important role in mitochondrial translation, which secondarily affects complex I assembly, of which many core subunits are mitochondrially encoded.^59^ Inherited in as an autosomal recessive pattern, clinical phenotype comprises the following: hypotonia, muscle weakness, muscle atrophy, exercise intolerance, developmental delay, neuroregression, and seizures. Muscle biopsy shows abnormal mitochondria, and brain imaging shows white matter lesions and pontocerebellar hypoplasia. Biochemically, affected individuals show hypoglycemia, lactic acidosis, and low complex I in the muscle.^60^-^63^

### 7. X-Linked sideroblastic anemia with ataxia due to ABCB7 gene defect (OMIM#301310)

The function of ABCB7 gene is to export a sulfur containing compound from mitochondria to the cytosol. Inherited in an X-linked recessive pattern, patients with defects in ABCB7 gene present clinically with ataxia and sideroblastic anemia in early childhood. Laboratory investigations show increased free erythrocyte protoporphyrin, hypochromic microcytic anemia, and ringed sideroblasts on bone marrow aspirate.^64^-^66^ Interestingly, heterozygous females have a normal neurologic examination and may have a dimorphic peripheral blood smear with both hypochromic microcytic anemia and normal red blood cells, and may additionally have ring sideroblasts on bone marrow examination.^67^

### 8. EVEN-PLUS syndrome due to HSPA9 gene defect (OMIM#616854)

EVEN-PLUS syndrome was described recently. The phenotype involves epiphyses, vertebrae, ears, and nose which are included in the EVEN part of the acronym, and PLUS associated findings include short stature, vertebral and epiphyseal changes, microtia, midface hypoplasia with flat nose and triangular nares, cardiac malformations. Other findings include anal atresia, hypodontia, and aplasia cutis. These features can overlap with findings reported in patients with Cerebral, Ocular, Dental, Auricular and Skeletal syndrome characterized by multiple congenital anomalies including Cerebral, Ocular, Dental, Auricular and Skeletal anomalies.^68^

Royer-Bertrand et al^69^ 2015 reported 3 girls from Korean and Chilean origins with EVEN-PLUS syndrome and confirmed the diagnosis with mutations in HSPA9 gene, a gene that is essential for mitochondrial protein import. While homozygous mutations in HSPA9 can cause EVEN-PLUS syndrome, heterozygous mutations can cause autosomal dominant sideroblastic anemia type 4.^69^,^70^

### 9. Lipoic acid biosynthesis defects

**Figure 2:**
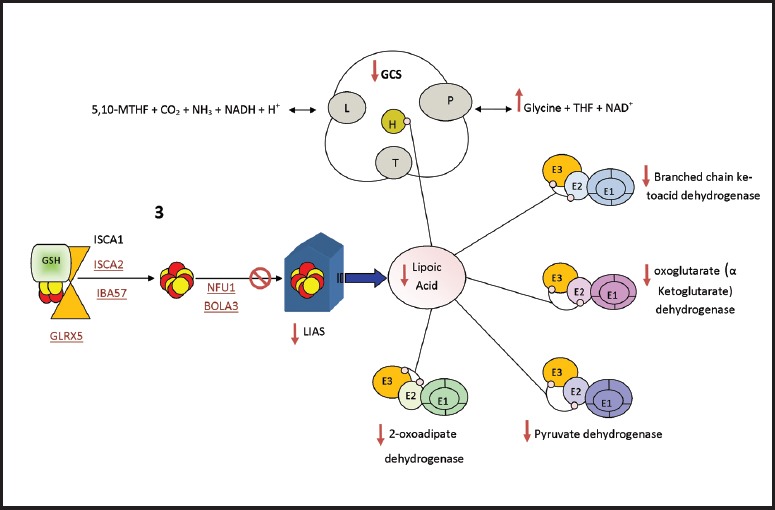
- Lipoic Acid Synthetase (LIAS) biosynthesis and function, LIAS is essential in the maturation of lipoic acid (LA) which acts as cofactor for many enzymes. lipoic acid is bound to E2 and E3 subunits of the mitochondrial a-ketoacid dehydrogenase complex family (Pyruvate dehydrogenase, oxoglutarate (a ketoglutarate) dehydrogenase and branched chain ketoacid dehydrogenase, 2-oxoadipate dehydrogenase). lipoic acid is also bound to protein H in the Glycine cleavage system (GCS). Diseases which cause inhibition of any of ISC assembly step 3 proteins (shown in red and underlined) will affect the synthesis of the target apoprotein LIAS and subsequently the end product LA. As a result, all the biological process which include lipoic acid will be affected; the activity of a-ketoacid dehydrogenase complex family will decrease and the GCS activity will decrease which result in hyperglycinemia. ISC - Iron–sulfur clusters, 5,10-MTHF - 5, 10 methylene tetrahydrofolate, GCS - Glycine Cleavage System, THF - tetrahydrofolate

One important biochemical phenotype of many mitochondrial ISCs biogenesis disorders is hyperglycinemia, which occurs due to defects in the third and final step that leads to inhibition of lipoic acid synthase which is a covalently bound cofactor essential for glycine cleavage system (GCS), an impairment of which results in hyperglycinemia (**Figure 2**). Lipoic acid is a vital cofactor for five redox reactions in humans: Two enzymes that are essential for energy production (a-ketoglutarate dehydrogenase, and pyruvate dehydrogenase PDH), and three that contribute to amino acid pathways (branched-chain ketoacid dehydrogenase (BCKDH), 2-oxoadipate dehydrogenase, and the GCS) (**Figure 2**). Lipoic acid biosynthesis defects were previously described in five syndromes: a) Multiple mitochondrial dysfunctions syndrome 1 due to NFU1 gene defect (OMIM#605711);^24^,^71^-^73^ b) Multiple mitochondrial dysfunctions syndrome 2 due to BOLA3 gene defect (OMIM#614299);^24^,^74^,^75^ c) Multiple mitochondrial dysfunctions syndrome 3 due to IBA57 gene defect (OMIM#615330);^76^-^78^ d) Multiple mitochondrial dysfunctions syndrome 4 due to ISCA2 gene defect (OMIM#616370);^79^ e) Non ketotic hyperglycinemia due to GLRX5 gene defect (OMIM# 616860 and 616859).^75^

These 5 disorders share one biochemical abnormality which is hyperglycinemia. The 4 multiple mitochondrial dysfunction syndromes have similar clinical and biochemical phenotypes which include: hypotonia, respiratory distress, seizures, encephalopathy, myopathy, neurologic regression, lethargy, poor feeding, optic atrophy, and diffuse white matter lesions seen on brain imaging. Furthermore, metabolic acidosis, lactic acidosis, increased urinary 2-hydroxybutyrate, and decreased activity of lipoic acid dependent enzymes including PDH, a-KGDH, BCKDH, and GCS. Additionally, muscle biopsy shows low complexes I and II.^24^,^68^,^71^-^75^,^78^,^79^

The last disorder due to GLRX5 gene defect has two clinical phenotypes: one is sideroblastic anemia, and the other constitutes childhood spasticity. However, biochemically, both phenotypes present with hyperglycinemia in the plasma of affected individuals.^75^

## Diagnostic approach

Mitochondrial ISC biogenesis disorders should be considered in the differential diagnosis when there is sideroblastic anemia, neurological manifestations or myopathic phenotypes, and follow up biochemical workup should be pursued. Hyperglycinemia, for instance, should point to lipoic acids biosynthesis defect disorders after excluding classical non ketotic hyperglycinemia caused by glycine decarboxylase deficiency, aminomethyltransferase gene, and glycine cleavage system H gene. Sideroblastic anemia with neurological manifestations could suggest GLRX5, ABCB7 or HSPA9 gene defects. Short stature, skeletal abnormalities and dysmorphic features could suggest EVEN-PLUS syndrome. One diagnostic approach for patients of consanguineous parents presenting with neurological or myopathic findings, is to consider homozygosity mapping and whole exome sequencing (WES) or whole genome sequencing.

## Future implications and conclusion

Prior to 2010, only a few human diseases related to mitochondrial ISC biogenesis defects were elucidated. With the introduction of next generation sequencing technologies, it has become more possible to expand these disorders and better characterize their clinical phenotypes, consequently more disorders are expected to be identified in the near future. The complexity and mixture of genetic and clinical heterogeneity (as illustrated by previous disorders) make ISC biogenesis defects difficult to recognize, and it is plausible that their prevalence is under estimated.

In conclusion, the combined clinical and advanced molecular diagnostic efforts will likely identify more disorders linked to the mitochondrial ISC biogenesis pathway, and potentially discover new genes in association with such diseases. Continued studies will help further elucidate mitochondrial iron homeostasis regulation, as understanding of mitochondrial iron overload could potentially yield better therapeutic approaches for such defects.
